# Combining paid work and family care for a patient at the end of life at home: insights from a qualitative study among caregivers in the Netherlands

**DOI:** 10.1186/s12904-021-00780-9

**Published:** 2021-06-24

**Authors:** Femmy M. Bijnsdorp, Bregje D. Onwuteaka-Philipsen, Cécile R.L. Boot, Allard J. van der Beek, Hanna T. Klop, H. Roeline W. Pasman

**Affiliations:** 1 Department of Public and Occupational Health, Amsterdam UMC, Vrije Universiteit Amsterdam, Amsterdam Public Health research institute, Expertise Center for Palliative Care, Amsterdam, the Netherlands; 2 Department of Public and Occupational Health, Amsterdam UMC, Vrije Universiteit Amsterdam, Amsterdam Public Health research institute, Amsterdam, the Netherlands

**Keywords:** Family care, paid work, life-threatening illness, end-of-life, interview study

## Abstract

**Background:**

Population ageing, an emphasis on home-based care of palliative patients and policies aimed at prolonging participation in the labour market are placing a growing demand on working family caregivers. This study aimed to provide insight into experiences with combining paid work and family care for patients at the end of life, factors facilitating and hindering this combination, and support needs.

**Method:**

Semi-structured interviews were held between July 2018 and July 2019 with 18 working family caregivers of patients with a life-threatening illness who were living at home. Transcripts were analysed following the principles of thematic analysis.

**Results:**

Some family caregivers could combine paid work and family care successfully, while this combination was burdensome for others. Family caregivers generally experienced a similar process in which four domains — caregiver characteristics, the care situation, the work situation and the context — influenced their experiences, feelings and needs regarding either the combination of paid work and care or the care situation in itself. In turn, experiences, feelings and needs sometimes affected health and wellbeing, or prompted caregivers to take actions or strategies to improve the situation. Changes in health and wellbeing could affect the situation in the four domains. Good health, flexibility and support at work, support from healthcare professionals and sharing care tasks were important in helping balance work and care responsibilities. Some caregivers felt ‘sandwiched’ between work and care and reported physical or mental health complaints.

**Conclusions:**

Experiences with combining paid work and family care at the end of life are diverse and depend on several factors. If too many factors are out of balance, family caregivers experience stress and this impacts their health and wellbeing. Family caregivers could be better supported in this by healthcare professionals, employers and local authorities.

**Supplementary Information:**

The online version contains supplementary material available at 10.1186/s12904-021-00780-9.

## Background

The care systems of many Western European countries, including the Netherlands, are under pressure due to population ageing. There is currently a shift to home-based care with the aim of curbing public expenditure and overcoming shortages in professional healthcare, and in response to people’s preference for living at home until the end of life [1, 2]. Care for patients living at home with palliative care needs often requires a substantial contribution from family caregivers [3]. Family caregivers provide unpaid care to relatives or friends, which can include nursing care, coordination of care, assisting with doctors’ visits, transportation, administrative support, household care or emotional support [4]. This care is partly provided by family caregivers who have paid work as well, particularly by adult children who provide care to their parents and by partners of people who are confronted with a life-threatening illness when relatively young [5]. Changing policies in care provision and policies aimed at prolonging participation in the labour market, such as an increase in the statutory retirement age, place a growing demand on working family caregivers. Due to these shifts, it is expected that more people will have to combine paid work and family care in the near future [6, 7].

In the Netherlands, formal care leave arrangements have recently been made available for workers that also provide family care. These arrangements include paid and unpaid leave for varying periods. For sudden, short episodes, there is the possibility of an emergency leave, which is a leave for few hours or days when a relative suddenly needs care, including full payment for at least one day. Short term care leave involves a maximum of twice the number of working hours per week over a period of 12 months. For example, workers with a 30-hour working week, are allowed 60 h of leave per 12 months. During this short term care leave, these workers are being paid at least 70 % of their salary. For longer episodes of family care, workers can take leave for a maximum of six times their number of working hours per week over a 12-month period. This leave is often unpaid. Even though there are different care leave arrangements available, these arrangements are hardly used by working family caregivers. The reasons for this remain unclear [8–10], while there seems to be a need for work adjustments.

A recent scoping review identified several challenges in combining family care and paid work, which included high or competing demands, psychosocial or emotional stressors, adverse health and financial pressure [11]. Family caregivers of a patient who is approaching the end of life are faced with the additional burden of coping with feelings of grief and loss, while they are often involved in intensive and complex caregiving tasks [12, 13]. Also, it has been found that family caregivers of patients near the end of life experienced more negative health outcomes compared to other caregivers [12]. One in five working family caregivers of patients with a terminal illness experienced a heavy burden [8], and a heavy burden was also found among employed caregivers of older cancer patients [14].

In addition, the care that is needed for people with a life-threatening illness can be quite unpredictable and differs between patients and illness progression trajectories [15]. This could result in uncertainty about the care that is needed and might make it more difficult to combine caregiving and work responsibilities [16]. Prior research has indicated that unpredictable care needs and emergencies were a source of competing demands and often led to absenteeism from work as the caregiver unexpectedly had to stay with the care recipient or leave work. Also, family caregivers were sometimes unable to concentrate at work, causing a decline in work productivity, because they were worried about the care recipient [11]. In this sense, providing family care for patients with a life-threatening illness could have consequences for their participation in the labour market.

Previous studies have shown that the majority of working caregivers caring for patients at the end of life adapted their work situation (e.g. reduced their working hours, quit their job, took leave or changed jobs) [8, 17]. Also, having to make a lot of work adjustments has been associated with worsening mental health of family caregivers [18]. To prevent this, it is crucial that working family caregivers are supported adequately.

Despite the growing attention paid to family caregiving in both the field of occupational health and the field of palliative care, research on the combination of paid work and family care for patients with a life-threatening illness is limited. Also, employers and occupational physicians often do not take the prognosis of the disease into account (e.g. curability), while this might be important for both the burden and the family caregiver’s capacity for work (e.g. they might be more emotional). In the field of palliative care, little attention has been paid to the context of work in understanding caregiver burden, whereas work could interfere with caregiving tasks. Because of this, working caregivers might experience more of a burden and more restrictions due to dual responsibilities. The aim of this study was therefore to provide in-depth insight into the experiences and support needs of working family caregivers of patients with a life-threatening illness, barriers and facilitators in the combination of work and family care, and how this affects family caregivers. A broader understanding about this is of relevance to healthcare professionals, employers and policy makers as it will help them offer timely support for family caregivers who provide end-of-life care to a relative or friend.

## Methods

### Design

Data was used from a longitudinal qualitative study, in which in-depth interviews were held to better understand the experiences and support needs of working family caregivers of patients with a life-threatening illness over time. The interviews are still ongoing and are being held in one to four rounds (situation as at October 2020). This study draws on the first round of interviews. The consolidated criteria guidelines for reporting qualitative studies (COREQ) were followed for the reporting on the qualitative data (see Additional file 1) [19].

### Recruitment and sampling

Participants were recruited by the researcher (FB) using two strategies: (1) Purposive sampling via general practitioners from different regions in the Netherlands. The general practitioners were asked to inform family caregivers about the study if they provided family care at home to a patient with a life-threatening illness and had paid work for at least twelve hours per week. Life-threatening illness was defined as a serious or advanced illness in which the patient cannot be cured and is approaching death (e.g. incurable cancer, chronic lung disease, heart failure, dementia, progressive neurodegenerative disorder). The general practitioners provided the family caregivers with a participant information letter. Maximum variation in the sample was sought in terms of gender, working hours, sector, illness type and intensity of care. (2) Convenience sampling via posters in several departments of a Dutch academic hospital and an item in the corporate newsletter of this hospital with information about the study and contact details of the researcher. Potential participants could contact the researcher and the researcher gave them the participant information letter.

Family caregivers who were willing to participate first completed an online questionnaire (see Additional file 2), including questions about their gender, age, education, work characteristics (e.g. employed or self-employed, working hours per week and sector) and characteristics of the care situation (e.g. relationship to care recipient, type of illness, type of caregiving tasks, intensity of care and whether the care recipient lived at home or in a residential home). The questionnaire could also be filled in on paper on request. Participants were included if they were at least 18 years old, provided at least one hour of family care per week to someone with a life-threatening illness who was living at home, and had paid work for at least twelve hours per week. Drop-out did not occur because family caregivers volunteered for participation themselves. The characteristics of the participants are presented in Table 1.

**Table 1:** Characteristics of participants (*n* = 18)

#	Gender	Age	Education	Employment	Sector	Work ^**a**^	Care ^**a**^	Care recipient	Type of illness	Duration of illness	Other caregivers involved	Contact frequency
1	Female	50-55	Middle	Employed	Health and social care	36	15	Parents (in-law)	Dementia / cancer, other ^*b*^	4 - 5 years	Family, home care staff, adult day care	Weekly
2	Female	35-40	Middle	Employed	Health and social care	16	12	Partner	Cancer	3 - 12 months	Family, district nurse	Lives in same house
3	Female	40-45	Middle	Employed	Health and social care	36	5	Parents (in-law)	Cancer / dementia	3 - 12 months	Family, home care staff	Daily
4	Female	50-55	Middle	Employed	Health and social care	24	25	Partner	Progressive neurodegenerative disorder	1 - 2 years	No	Lives in same house
5	Female	55-60	High	Employed	Business	39	14	Parents (in-law)	Dementia, stroke, other / organ failure, other ^*b*^	1 - 2 years	Family, home care staff	Daily
6	Male	50-55	Middle	Employed	Health and social care	20	30	Partner	Organ failure; dementia; other ^*b*^	> 5 years	No	Lives in same house
7	Female	60-65	Middle	Employed	Public services	18	90	Partner	Progressive neurodegenerative disorder; organ failure	> 5 years	Family, household help	Lives in same house
8	Female	55-60	Middle	Employed	Health and social care	32	8	Parent (in-law)	Dementia; cancer	1 - 2 years	No	Daily
9	Female	50-55	High	Employed	Health and social care	28	35	Partner	Cancer	< 3 months	No	Lives in same house
10	Female	40-45	Low	Self-employed	Creative arts	40	15	Parents (in-law)	Dementia / stroke	1 - 2 years	Family, 24 hours home care	Daily
11	Male	65-70	High	Employed / Self-employed	Business + FMCG c	12	28	Partner / parent (in-law)	Stroke / organ failure	> 5 years	Home care staff, household help	Lives in same house
12	Male	40-45	High	Employed	Education	40	16	Parent (in-law)	Cancer	2 - 3 years	Family	Daily
13	Female	55-60	High	Employed	Education	24	10	Parent (in-law) / other family member	Cancer / dementia	1 - 2 years	Family, household help, volunteer	Weekly
14	Female	35-40	High	Employed	Health and social care	24	50	Child	Cancer	3 - 12 months	Family	Lives in same house
15	Female	50-55	High	Employed	Public services	28	25	Partner	Progressive neurodegenerative disorder	1 - 2 years	Family, home care staff	Lives in same house
16	Female	50-55	Middle	Employed	Health and social care	20	10	Parents (in-law)	Progressive neurodegenerative disorder / other ^*b*^	3 - 12 months	Family	Daily
17	Male	30-35	High	Employed	Public services	36	12	Parent (in-law)	Cancer	2 - 3 years	Family	Daily
18	Male	30-35	High	Employed	Public services	36	15	Partner	Cancer	2 - 3 years	Home care staff	Lives in same house

Notes: ^a^ in hours per week; ^b^ other included: rheumatism, psychiatric disorder, frailty, disability; ^C^ Fast-moving consumer goods

### Setting and data collection

Semi-structured interviews were held with 18 family caregivers between July 2018 and July 2019, and were guided by a topic list (see Additional file 3). The interpretations of the researcher were checked by the participant after summarizing the most important themes during and at the end of the interview. The interviews were held at the participant’s location of choice, which was often their own home, their workplace or the premises of VU University in Amsterdam. All interviews were audio-recorded and conducted by one female researcher who was trained in in-depth interviewing (FB). The duration varied between 45 and 120 min. After the interview, ad hoc field notes and a summary of the conversation were made to provide a first impression of the content. A summary of the results was returned to the participants for comment and/or correction. Data was collected until no new themes emerged from the data.

### Data analysis

All interviews were transcribed verbatim and were analysed in Atlas.ti 8 following the principles of thematic analysis [20, 21]. Data from the first five interviews was coded inductively and discussed by FB, HRP and HK. Discrepancies in the coding were discussed until consensus was reached. Some topics related to communication needed to be highlighted more in the following interviews and were added to the topic list. The remaining transcripts were analysed and coded by FB. Also, an across-case analysis was conducted, in which all text fragments associated with the code were explored to provide a broader understanding of the content of the codes [22]. The emerging codes were grouped into overarching themes and integrated into a framework to illustrate the relation between the themes. Interpretations, themes and the framework were discussed regularly with all team members. The framework was validated deductively by FB by rereading all transcripts and constantly comparing the framework with the transcripts. This led to additional changes to the framework, including the adaption of strategies and the impact on emotional and social wellbeing.

## Results

**Fig. 1:**
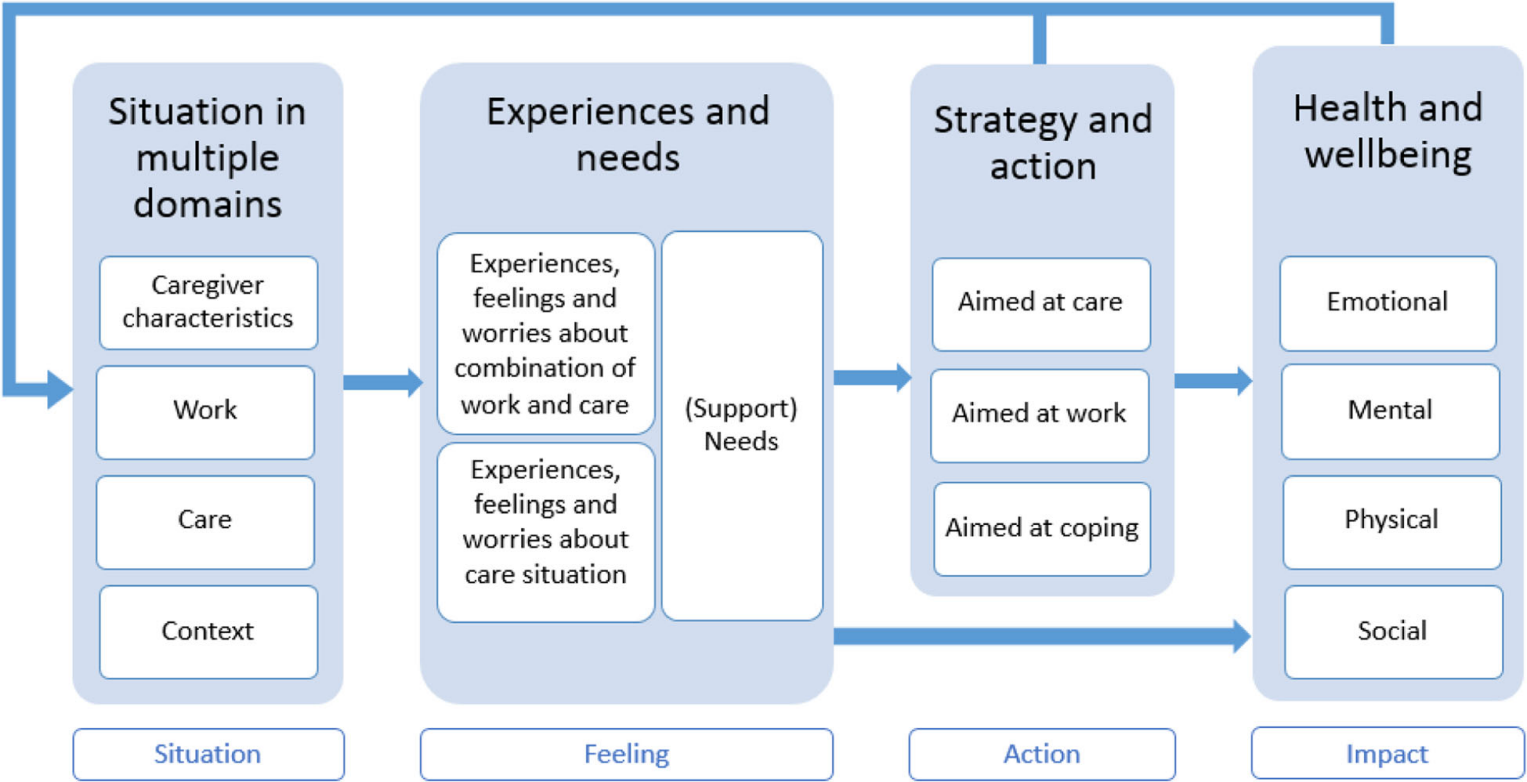
Framework of experiences and impact combination work and care for patients with a life-threatening illness

The family caregivers who were interviewed generally experienced a similar process in which four domains, namely characteristics of the caregiver, the care situation, the situation at work and the context, influenced their experiences, feelings and needs regarding either the combination of paid work and care or the care situation in itself (see Fig. 1). Examples of caregivers’ characteristics were the caregiver’s own health, their attitude towards caregiving, their perceived caregiving skills, their feeling of responsibility, their wish for control and the ability to set boundaries. The care situation included the relationship with the care recipient, the illness trajectory, prognosis and care intensity. The situation at work included working hours, support and understanding from the supervisor and colleagues, autonomy and flexibility. The broader context covered aspects such as the ability to share care tasks with others, and communication with healthcare professionals, municipalities or other organizations.

In turn, experiences, feelings and needs sometimes affected the caregivers’ health and wellbeing, or they initiated certain actions or strategies to improve the situation. For some caregivers, the adopted strategies could prevent deterioration in their health and wellbeing, and could influence the situation in multiple domains such as work or care provision. Likewise, changes in health and wellbeing could affect the situation (or the perceived situation) in these multiple domains. Table 2 illustrates the framework by relating two stories of working family caregivers looking after patients with a life-threatening illness. To ensure privacy, names and some characteristics have been changed. In the following section, the themes within the framework are explained in detail.

**Table 2:** Illustration of the framework

Caregiver story	Integration into the framework
James is 57 years old, works part-time in the health and social care sector and provides care for his wife, who suffers from a progressive neurological disorder and dementia. James finds the combination of paid work and family care burdensome. Because of the progressive neurological disorder and dementia, his wife’s behaviour has changed and she is sometimes aggressive and unpredictable. James cannot share the care tasks with others because his wife does not accept help from others. When James is at work, he often receives phone calls from his wife, which distracts him sometimes. James has a senior position, but he wants less demanding tasks and does not want to work on weekends anymore because it is too much to combine this with the care for his wife. James discussed this with his supervisor, but there was no understanding for his situation and it was not possible to adapt his work situation. He feels pressured, he experiences a psychological burden and the situation costs him a lot of energy. When James starts experiencing physical complaints because of the stress, he decides that changes have to be made. He cannot change the situation at home because his wife does not accept help from others. Therefore he feels that his only option is to find another job.	Situation: For this family caregiver, the barriers are mainly at work (e.g. demanding tasks, working in weekends, no understanding from his supervisor, no arrangements to adapt his work) and in the care (e.g. the care recipient claims the family caregiver and does not accept help from others). Experiences and needs: Because of these barriers the family caregiver experiences the combination of work and care as burdensome. Strategies and impact on health and wellbeing: His first strategy (discussing his situation with his supervisor) did not have the desired result as no arrangements could be made. This impacted his mental and physical health, which made the situation even more difficult. Then he adopted another strategy (searching for another job), which will impact his work situation if he changes jobs and will probably improve his health and wellbeing.
Emma is 52 years old, works fulltime in the financial sector and provides care to her father, who was diagnosed with incurable cancer three months ago. The prognosis is bad and they have started chemotherapy to prolong his life and ease symptoms. Emma expects that her father will not be around for much longer. She is determined to provide the care he needs until the end, together with her sister. Emma and her father have a close relationship and can talk about everything. Her father indicated that if the care becomes too intensive for her, he wants to go to a hospice. Knowing this gives Emma confidence that she will be able to cope with the situation. Emma has informed her supervisor and colleagues about her father’s situation. They support her by listening and they help find solutions if necessary. Emma can be flexible in scheduling work appointments and can easily work remotely. Sometimes she works on her laptop in the hospital when her father is receiving chemotherapy. Emma likes her job and working provides respite from care. Because of the social support she receives at work, Emma does not experience barriers in the combination of work and care and therefore experiences no impact on her own health. However, knowing that she will lose her father soon makes her feel very sad and frustrated. For her the grieving process has already begun.	Situation: This family caregiver experiences few barriers in the combination of care and work since she has resources from work (e.g. social support, flexibility at work, ability to work remotely and respite from care). Also, characteristics of the care situation (e.g. the close relationship with the care recipient, good communication with the care recipient, limited expected duration in which care is needed and the option to transfer her father to a hospice if necessary) and context factors (e.g. sharing care tasks) help her cope with the situation. Experiences and needs: This caregiver can successfully combine work and care and does not find the combination difficult. Health and wellbeing: The situation is emotionally demanding due to the impending death of her father. Thus, her experiences have some impact on her health and wellbeing.

### Impact of multiple domains on experiences, feelings and worries about combining work and care

#### Keeping a balance between paid work and family care

Some family caregivers felt they could combine work and care successfully. Key facilitators for keeping a balance between work and care demands were being able to set boundaries, sharing care tasks with family or friends, support and understanding from supervisors and colleagues at work, having autonomy at work, flexibility in working hours and being able to work remotely (Table 3, Q1). Supervisors and colleagues in particular played an important role in how family caregivers experienced the combination of work and family care (Table 3, Q2). Having short lines of communication with healthcare professionals helped family caregivers to make decisions effectively and plan ahead, which made the combination easier. Also, it was less complicated to combine work and care if there were few unpredictable, acute situations with the care recipient.

**Table 3:** Quotes

No.	Theme	Framework	Quote
Q1	Flexibility at work	Work	*I think that it’s precisely… well… that flexible way of working, so that if you need to go to the hospital in the afternoon, you first do a few hours at home in the morning; you sort it out again that way. (#7)*
Q2	Support at work	Work	*There’s a lot of understanding for my situation. My boss also looks at what tasks I have and whether we should be reassigning some tasks. And my co-workers regularly ask how things are going and if I’m coping, and they say they’re amazed that I’m still turning up despite all my care stuff that I’m doing at home. Yes, that feels good. (#14)*
Q3	Juggling responsibilities	Experiences and needs	*If you have a 36-hour working week and you’re also spending your entire Saturday there [with the care recipient], then you only really have the Sunday to yourself. And then you often have engagements, birthday parties or things to do; then it’s Monday again and you’re basically exhausted just as you have to go back to work again. (#3)*
Q4	Support needs at work	Experiences and needs	*Well, a few more options. I have four care leave days a year and that’s just not enough. I think that should definitely be increased a bit; it costs me a lot of money too. I work in the care sector but my feeling is that they don’t care that much for the staff. […] Show that you know they’re there and that you appreciate the fact that they are always there despite everything. For example, I never turn up late to work. So show some appreciation; just a bit of attention and a pat on the back every now and then would help hugely. (#6)*
Q5	Flexibility from healthcare professionals	Context	*I try to plan the doctors’ visits to fit in with my work so that I don’t have to take leave but that’s not always possible. Not by a long way. That’s also one of my frustrations, that organizations just... For example, he has to go to the hospital once every eight weeks for an infusion. So you need to be there at one thirty or two o’clock, but if I work until one, I want to have a bite to eat, let the dog out, then there’s the travelling time... so I always try and ask whether it could be a bit later. “No, because there’s no doctor available then.” Or you try to schedule an examination... No, they say, because we don’t yet know the doctor’s timetable, and you’re talking about the next month. Then I think: come on, how about a different doctor perhaps... just schedule that appointment. So I have to make phone calls again to arrange it. These are often the issues you run into. (#7)*
Q6	Communication with municipalities and other organizations	Context	*They’d lost the application we’d given the municipality’s care office, so I had to apply for everything all over again. Separately for each component, at that. And I’m talking about the stairlift, the shower chair, the mobility scooter, extra public transport… So at one point I asked, ‘Hey guys, can’t you do one big intake, because everything’s noted down there – we did an intake for the extra public transport where we discussed everything, including the home situation, so everything is recorded there.’ Couldn’t they just use that for the mobility scooter and the shower chair and the stairlift? “Yes, that might be a good idea.” I never heard anything more about it. (#6)*
Q7	Assigned contact person	Experiences and needs	*[The care recipient] was being treated at [the hospital] and I reckon they’re very good in terms of the care they give, in fighting cancer and explaining it all. But they don’t do anything extra in addition to that. And it’s quite difficult to know where you should go if something is up or who you can contact just to discuss things. Then I heard on the grapevine about the Social Support scheme, so we applied for that and the woman there was able to give us some more tips. Well anyway, we are trying to find a little bit of help, very slowly, step by step, but it would be ever so nice if there was a single contact point where you could go. Someone who could help you. There isn’t really that option, not in our experience, and I find this makes things difficult. (#12)*
Q8	Communication with healthcare professionals	Experiences and needs	*At the start, with the ‘bad news’ talk, I had to do a lot of chasing up with phone calls. There was a miscommunication with the hospital: “I’ll phone you the next day with the result”, and then they didn’t call. “Oh yes, I’ve got another blood test I didn’t pass on”, so I had to phone up about that. The lungs of [the care recipient] were filling with fluid, so I phoned the [hospital] again. Well, I spent three days chasing up that on the phone. […] So you’re basically spending all your time on medical matters, but then you have to phone another hundred people and wait for answers. Of course, they performed very badly in informing us. I also think that if you’ve sent someone home after giving them bad news, that someone – a nurse – should phone them and check how it’s going: “Do you have any questions about that talk? You could also set various wheels in motion if necessary...” None of that happened. I had to figure it all out for myself. (#2)*
Q9	Frustration in relation to illness progression	Experiences and needs	*The frustrating thing is that I’m standing there next to him and I see it but I can’t do anything. He’s in pain, but I can’t make the pain any less, I can’t help him, I can’t do anything, all I can do is watch that person deteriorating. There’s no point in being sad because then you... I think it’s a taboo for a family caregiver, you’re not allowed to complain about how hard it is for you, or to say that you feel sad or need to cry; you don’t do that because you’re not the one dying. Right? (#4)*
Q10	Grieving process	Experiences and needs	*Yes, that was a real ‘bad news’ talk. They were very clear in [the hospital], very honest, very good. And we looked one another in the eye, kind of this is it, this is the beginning of the end. Right, that day... I won’t easily forget where we were then and what happened. Then you hear what you’ve been afraid of, you know it’s going to come eventually but you hope it will be a long time before it does. Then the process of saying goodbye to one another starts; and that’s when the grieving starts too. (#18)*
Q11	Sense of fulfilment	Experiences and needs	*It gives fulfilment and it makes you feel better; it might also be part of the process of saying goodbye, guiding you towards saying goodbye. You spend an awful lot of time together; you’re literally caring for someone. [...] Yes it’s tough, but I’m also grateful for this. (#12)*
Q12	Sharing care tasks	Strategy and action	*And [the sister-in-law] also cooks for her sometimes and she accompanies her to doctors’ appointments and to the shops, so she’s doing an awful lot. I can’t bear to think about it if she were to say she didn’t want to do that anymore. I wouldn’t know what to do then. Well, you’d have to have a stranger come along. I mean, I’m not going to give up my job — I won’t do that. No way. Well anyway, she’s still doing it for the moment and she does it willingly. [...] So we have really welcomed that; I definitely wouldn’t want to be without it. (#5)*
Q13	Compassionate care leave	Strategy and action	*Well, that’s the tricky thing, you know. Of course, where we work [works in a hospital] there are lots of people with cancer; there comes a point when it’s a kind of final stage. So you could say “I’ll take care leave” but I can’t do that now because it could still be another two years... and I need the money too. But well, you know, it’s difficult. And as it gets worse, I’ve no idea how you’re supposed to combine that with your work. (#1)*
Q14	Coaching programme	Strategy and action	*At the start of the year, I was pretty much at my limit, so I started a coaching programme and that helped me to cope with it. I was starting to get fairly stressed out. [...] I was on a short fuse and simple things started to cost me so much energy. So then I told [my boss] and I got a lot of support there.I do feel much stronger now; I feel I can manage it at the moment. (#12)*
Q15	Experienced burden due to combination of work and care	Health and wellbeing	*I’m out of balance, things are tough at home, it’s not going well at work and the scales are tipping. I’ve really lost that balance completely. [...] I notice I’ve got these physical complaints, shortness of breath, hair loss, not eating properly, sleeping badly. My back often goes – I’m exhausted, I’m finished. (#4)*
Q16	Importance of good health and sleep	Health and wellbeing	*That I really do get a good night’s sleep, because I do notice that... if I’m really at it twenty-four hours a day or if I have to get up in the middle of the night, that genuinely gets to you. All sorts of things were going on in that first period. Once I had to get up in the night four times in one week. Well, you can’t keep that up, it absolutely wears you out. So I’m pleased that that [assistance from the 24-hour nursing service] is going to be starting now as it will help me keep going, give me a bit more rest. (#10)*
Q17	Financial impact	Health and wellbeing	*I’ve had to give up a lot in financial terms. [...] I reduced my working hours, for example, and that cost me a lot of money. All the patient’s contributions that we have to pay soon add up. Well, I think... right, if you can show that you cut your working hours for that reason, and I still earn a pretty good salary, but well… if I then have to spend it all on various facilities, of course it’s pretty galling, and that’s not what I studied for or what I do my job for: I do it so that I can live off the money, not give it away. (#6)*

Other caregivers struggled with the combination of paid work and family care, and experienced this as burdensome. In general, a lack of the aforementioned facilitating factors hindered the combination of work and care, and increased the burden. Some caregivers found the combination of work and care difficult because they had to juggle so many responsibilities and hardly had time to rest (Table 3, Q3).

#### Relation between experiences with combination of work and care and (support) needs

Experiences, feelings and worries were often related to needs in care, at work or for their own health. For instance, caregivers who experienced barriers in their work often wanted more appreciation, support and guidance at work to help them find out what arrangements were available for them (Table 3, Q4). Some caregivers who experienced barriers in the care situation wanted to share care tasks with others more often, but were sometimes unable to arrange this. Others wanted more flexibility from healthcare professionals in scheduling care appointments (Table 3, Q5).

### Impact of domains on experiences, feelings and worries about the care situation and illness trajectory

#### Challenges in arranging care and support

In some cases, caregivers experienced consequences of a staff shortage in professional healthcare for the care recipient (e.g. long waiting lists), which put more pressure on them. Difficult communication with municipalities or other organizations proved to be an important theme. Problems included bureaucracy, lots of paperwork, unclear application procedures and files that went missing, which made it hard for caregivers to arrange the care that was needed (Table 3, Q6). This was time-consuming and strenuous for them, on top of all the other responsibilities in work and care. For many family caregivers who experienced this ‘struggle’ with organizations, the care they were entitled to and the support options were unclear. They had trouble arranging support and would have liked proper information and guidance in finding help (Table 3, Q7). Several caregivers were not satisfied with the communication between them and healthcare professionals, since they had to make a lot of phone calls themselves to find out medical results or treatment options (Table 3, Q8). In some cases, family caregivers felt there was a lack of aftercare from healthcare professionals when curative treatments stopped, and they wanted to be guided to the next step.

 In addition, when a relative needed care, daughters and sisters were more inclined to take up care responsibilities than sons or brothers. This sometimes caused tension between family members, since the women wanted to share their caregiving tasks more with their siblings. This was, however, difficult to accomplish, since (according to the caregiver) their siblings often did not have time to help, felt less responsible to provide care, or had other ideas about what would be suitable care (e.g. moving care recipient to a nursing home rather than providing intensive care at home).

#### Challenges related to illness progression

An unpredictable illness trajectory with acute exacerbations was identified as challenging, and family caregivers were unsure about what to expect in the future. This made it difficult to determine when to involve more (professional) care or when to take compassionate leave at work. Some caregivers felt frustrated, because they could not really help the care recipient, and could only watch their health decline (Table 3, Q9). Anticipatory grief was an important theme, as many caregivers said that they were already thinking about the impending death of their relative, and the corresponding grieving process had already begun (Table 3, Q10). For them, the situation was still emotionally demanding, even if they could combine work and care successfully. However, some caregivers indicated that taking care of their relative also gave a sense of fulfilment (Table 3, Q11). Caregivers found it difficult sometimes to cope with the fact that the care recipient’s behaviour had changed. This was identified as a problem in the case of patients with dementia or neurodegenerative disorders. In some cases, caregivers stated that it was important for them or for the care recipient for care to be provided at home until death. This sometimes put more pressure on the family caregiver, since a nursing home or hospice was then not an option.

### Impact of experiences and needs on strategies and action

Negative experiences and unmet needs caused the caregiver to initiate certain actions or strategies to improve the situation. Broadly, these strategies focused on three domains: changes in the care situation, changes at work, or improving their own ability to cope with the situation.

#### Strategies to improve the care situation

Strategies aimed at the care situation included arranging respite care, reaching out to other family members or friends to share care tasks, involving volunteers or privately paid care, or upscaling professional healthcare provision. For some caregivers it was not easy to involve other people in the care situation since the care recipient did not accept this. Several caregivers said that they did not want their paid work to suffer from the care situation and they tried to schedule or reschedule care appointments around their work schedule, or share care tasks with others (Table 3, Q12).

#### Strategies to improve work situation

Strategies aimed at work were adjusting working hours, taking compassionate care leave or taking unpaid leave. Notably, some family caregivers were unaware of the compassionate care leave options. Others did not take compassionate leave because they found it difficult to determine when they should take it, since working caregivers are only entitled to a limited number of days per year. They did not want to ‘waste’ the compassionate leave, because the care demands were unpredictable and the future was uncertain, or the financial impact would be too large (Table 3, Q13). Several family caregivers, mostly caregivers looking after a child or partner, expressed the view that the current compassionate leave scheme was not sufficient for their situation. Caregivers sometimes used other leave schemes to provide care, such as parental leave or vacation leave on a structural basis. Others saved vacation leave hours for acute care situations. Many caregivers made personal arrangements with their supervisor, including alternative or less demanding tasks, working more from home and flexible working hours, or they were referred to occupational health professionals. Less often, caregivers made arrangements with colleagues, such as trading shifts or taking over shifts. For some caregivers it was important to remain active at work, and work provided respite from the care situation. Several caregivers expressed the view that the care situation was more important to them than work and they tried to schedule or reschedule meetings at work to fit around care demands. In some cases, caregivers were searching for another job because they were unable to reconcile the current work and care demands. Sometimes caregivers explored whether early retirement was a solution, but often this was not feasible because of the financial impact.

#### Strategies to improve coping ability and mental health

Family caregivers sometimes sought help from a psychologist (e.g. cognitive therapy) or enrolled in a coaching programme (e.g. corporate welfare training or a lifestyle coach) (Table 3, Q14). In some cases, family caregivers practiced mindfulness or yoga, or went cycling or on evening walks to clear their mind, which enabled them to cope better with the demands of work and care. Others found an outlet in choir singing, or talking to family, friends or other caregivers, which could relieve tension. Many family caregivers tried to schedule free time for themselves to relax, but it was often difficult to stick to the time off they had set for themselves.

### Impact of experiences and strategies on health and wellbeing and the situation in different domains

As shown in the framework, experiences and needs could impact the health and wellbeing of family caregivers. This was especially the case when family caregivers found the combination of work and care burdensome. They felt listless and tired, had a short temper, were often in a bad mood and the combination cost them a lot of energy. Some caregivers said that their life was no fun anymore and that the situation was overwhelming them. They experienced burn-out symptoms or physical complaints such as shortness of breath, tiredness, a need for antidepressants, panic attacks, headaches or crying episodes (Table 3, Q15). Getting enough sleep and having good health themselves proved to be important in enabling them to cope with the situation, while many caregivers worried a lot and were not able to get sufficient sleep (Table 3, Q16). For the majority of caregivers, the care situation impacted their social life and involvement in the community. They felt lonely because they had no time for their friends, hobbies, volunteer work or relaxation because of the care and work demands. Several caregivers mentioned that the situation also impacted their financial situation (Table 3, Q17).

The strategies they adopted to improve the situation could directly impact work or care provision, for instance if caregivers reduced working hours or shared care tasks with others. If the strategies were successful, caregivers experienced less or no impact on their health and wellbeing. If the strategies did not have the desired results, their health and wellbeing were sometimes negatively affected. Declining health and wellbeing, in turn, often influenced the situation in the different domains as well. For example, if caregivers experienced burn-out symptoms due to the combination of work and care, work was impacted if the caregiver then searched for another job, or the care situation was affected as the caregiver was no longer able to perform all the care tasks.

## Discussion

This study provides in-depth insight into the experiences and needs of working family caregivers of patients with a life-threatening illness, the barriers and facilitators in the combination of work and care, and how this combination affects family caregivers. The experiences, needs and impact on health and wellbeing were found to be very context-specific. Barriers in one domain (e.g. a lack of flexibility or insufficient arrangements at work) could be compensated by resources from other domains (e.g. sharing care tasks with others). However, when there were too many barriers and insufficient resources to overcome them, caregivers experienced the situation as burdensome. This was often problematic for their health and wellbeing if they could not adopt strategies to improve the situation. In turn, this could affect work and care, and subsequently further impact experiences and support needs.

### Caregiver’ experiences, challenges and facilitators

In line with earlier findings [8, 23], experiences with the combination of paid work and family care for a relative with a life-threatening illness were not solely positive or negative, and did not depend on characteristics of work and care only. Even when caregivers could combine work and care successfully, the situation could still be burdensome or demanding. Anticipatory grief and emotional distress seemed to play an important role here, and in line with other research [24], often came with uncertainty about the illness, feelings of disruption of one’s own life, and a changing relationship with the care recipient. Aside from this anticipatory grief, challenges and facilitators in combining work and care were quite similar to what has been identified among a broader population of working family caregivers. This included for instance high and competing demands from work and care, unpredictable care needs, work interruptions, travel distance and inflexible schedules, which could be compensated by sharing care tasks, making work adjustments or receiving support at work [11, 25, 26]. When the care recipient required intensive caregiving, the illness progression was unpredictable with acute exacerbations, and/or when the prognosis of the care recipient was bad, family caregivers often experienced a negative impact on their mental health. This was especially found when other circumstances were also unfavorable, such as few support options at work. If these unfavorable conditions were present for a longer period of time, it was more difficult for caregivers to cope with the situation.

### Importance of the workplace

In accordance with prior research [25, 26], support from supervisors and colleagues in the workplace was critical in how the caregivers in this study experienced the situation. Negative attitudes or limited understanding of the caregivers’ situation contributed to conflict at work [11], sometimes forcing them to quit their job. On the other hand, work provided respite from the care situation for many caregivers. Consistent with earlier findings, this indicates that remaining active in the workforce is meaningful for family caregivers, as it could facilitate psychological respite and contribute to wellbeing [27]. Hence, it is key that family caregivers are supported in the workplace in such a way that they can retain a balance between work and care. Future research could further assess how communication about family caregiving at work takes place, and what supervisors need to do to give caregivers the support they need.

### Comparison between framework and existing models

The themes that were integrated into the framework share a number of commonalities with existing models that define important factors for family caregivers’ mental health. There are some clear similarities between the factors that proved to be important in our framework and the components of the Informal Care Integrative Model (ICIM) [28], which builds on the Model of Carer Stress and Burden [29] and the Job Demands-Resources model [30]. The ICIM states that caregiver characteristics, caregiving setting and social environment determine the subjective evaluation of balance or imbalance between demands and resources. This evaluation could then mediate between the consequences of the determinants and burn-out [28]. This is comparable to the situation in the multiple domains (i.e. caregiver characteristics, work, care and context) that relate to feelings and experiences in the current framework, which could in turn impact the health and wellbeing of the caregiver. In addition, the ICIM states that burn-out and physical or psychological issues could impact aspects of the caregiving context or social environment. Gérain and Zech [28] pointed out that these feedback loops have often been neglected in prior models, while it is critical to address the development of strain over time. These feedback loops were also incorporated in the current framework, where caregiver health and wellbeing could influence the situation in multiple domains.

To our knowledge, this was one of the few studies that provides in-depth insight into the experiences, challenges and facilitators of family caregivers for patients at the end of life in combination with paid work. The framework adds to academic knowledge about family caregivers at the end of life by showing that work characteristics and occupational demands and resources were important in determining experiences, needs, health and wellbeing. Also, we found that certain actions or strategies could mediate between experiences and health and wellbeing, or could modify the various domains. Future research could examine trajectories of experiences when combining work and care for a relative at the end of life and identify factors that influence this over time.

### Methodological considerations

Using semi-structured interviews provided rich and detailed information about the current situation and experiences of working family caregivers of patients with a life-threatening illness. Moreover, the rich data prompted the development of our framework. This framework is partly in line with existing theory, but also provides new insights, which is a strength of this study.

Although we included all male caregivers who responded, the experiences of men combining paid work and family care might be underrepresented. The results did, however, not indicate clear differences in how male and female caregivers experienced the combination of work and care, in the strategies that they used to improve the situation, or the impact of the situation on mental health and wellbeing. In addition, at the population level, women are more often involved in family care than men [5, 31]. However, in further research it might be good to give more emphasis to (including) male caregivers in order to perform appropriate gender analysis, since caregiving seems to be a women’s unpaid job.

Family caregivers with a non-Western cultural background were also underrepresented, and the role of cultural norms and values in the experiences of these caregivers may have been overlooked as a result [28, 32]. Furthermore, the family caregivers in the study generally worked in jobs with considerable autonomy, enjoyed flexibility at work and/or did not work fulltime. Caregivers working fulltime in jobs with little autonomy or with a lack of flexibility at work possibly did not have time to participate in the current study, while they might experience more difficulties with the combination of work and care. The majority of caregivers in this study worked in sectors with a high emotional workload (e.g. health and social care, and education), which might also have contributed to more emotional strain [33]. Caregivers with fulltime jobs and a lack of autonomy or flexibility deserve more attention in future research, since their experiences and challenges might be different. Also, although the educational level and job sector of the family caregivers provided some insights into the socio-economic position, specific information about income was not available. Since some caregivers noted that the financial impact of formal care leave arrangements would be too large, future research could also take the level of income into account.

The inclusion via general practitioners could on the one hand be considered a strong point, as they have information about patients with a life-threatening illness and are often also in contact with the family caregiver. However, on the other hand, this could also have contributed to selection bias, since they might have been more hesitant to invite family caregivers of patients in intensive care situations. The caregivers who provided the most hours of family care had applied to take part in this study themselves after they had seen the poster in the hospital. This suggests that even family caregivers in intensive situations are sometimes willing to participate in research and share their experiences. In addition, although we were interested in the experiences of family caregivers themselves, it should be considered that the results of this study are from a single stakeholder perspective. Future research could triangulate information from general practitioners and employers to increase the study’s comprehensiveness and validate the study results.

### Implications for healthcare professionals, employers and policy makers

This study indicated that there were some commonalities in support needs that have implications for policy and practice. Many working family caregivers of a relative with a life-threatening illness experienced anticipatory grief and emotional distress, which influenced their mental health in an adverse way. More psychological support might be needed to increase their emotional preparedness, and to guide them through the process until or after bereavement [34]. This fits the palliative care approach which is directed at supporting not only patients, but also their families [35]. This support need could be signalled by the healthcare professionals who are involved in the care trajectory of the care recipient. They could also advise caregivers about respite options, since many caregivers hardly had time to rest. In addition, healthcare professionals could improve guidance and aftercare when there are no more curative treatments by discussing questions and worries [36]. Increasing collaboration between family caregivers and professionals, and more involvement in planning care could help family caregivers to combine their care tasks with work demands.

In the workplace, supervisors could be better informed about the support options for family caregivers in their organization and how to deal with these situations, as understanding and open communication were experienced by caregivers as very supportive. Employers could provide more guidance about the available support options, such as compassionate care leave or other work arrangements, since family caregivers often had to find out themselves. Also, employers could facilitate consultation hours with an informal care expert, who could assess the caregiver’s situation in multiple domains, identify solutions for unresolved issues, and arrange respite care or for administrative tasks to be taken over temporarily. This support could help family caregivers to feel better able to combine work and care, and could prevent burn-out and long-term absence from work. Also, compassionate care leave arrangements warrant more attention by policy makers, since family caregivers were hesitant to use this type of leave. Family caregivers of patients with an unpredictable illness trajectory often found it difficult to determine when to take this leave because of its limited nature. Hence, it could be that the people who need this type of leave the most are not able to use it as they don’t want to ‘waste’ it when they might need it for worse times ahead. In addition, for some family caregivers it was financially not feasible to take compassionate care leave, since they would receive less or no financial compensation and the impact on their income would be too large.

Lastly, local authorities and health insurance companies could improve accessibility and application procedures, since many family caregivers experienced communication difficulties. They could increase awareness and advice about support services, as some caregivers were unaware of the available options. Policy makers should re-evaluate and simplify application procedures and provide information that is understandable for the general public. This could reduce feelings of pressure amongst caregivers and a sense that they are in a fight with organizations.

## Conclusions

Experiences with combining paid work and family care at the end of life are diverse and depend on several factors. If too many factors are out of balance, caregivers experience stress and this impacts their health and wellbeing. To facilitate a successful combination of paid work and family care, flexibility and understanding at work, timely support from professional caregivers or support services and/or sharing care tasks with others are important. Family caregivers could be better supported in this by healthcare professionals, employers and local authorities.

## Supplementary Information

**Additional file 1.** COREQ checklist. Consolidated criteria for reporting qualitative studies (COREQ): 32-item checklist.

**Additional file 2.** Questionnaire. Questionnaire that was used for background characteristics family caregivers.

**Additional file 3.** Topic list for semi-structured interviews. Topic list that was used in this study.

## Data Availability

The data used during the current study are available from the corresponding author on reasonable request.

## Abbreviations

COREQ: consolidated criteria guidelines for reporting qualitative studies
FB: author Femmy Bijnsdorp
HRP: author H Roeline Pasman
HK: author Hanna Klop
FMCG: Fast Moving Consumer Goods
ICIM: Informal Care Integrative Model
