# Supplementary material for: Combining paid work and family care for a patient at the end of life at home: insights from a qualitative study among caregivers in the Netherlands
Source: BMC Palliat Care. 2021 Jun 24;20:93. doi: 10.1186/s12904-021-00780-9 (PMC8228921; doi:10.1186/s12904-021-00780-9)
Supplement: Supplementary file 1 — Additional file 1. COREQ checklist. Consolidated criteria for reporting qualitative studies (COREQ): 32-item checklist. [file 12904_2021_780_MOESM1_ESM.docx]

**Additional file 1 – Consolidated criteria for reporting qualitative studies (COREQ): 32-item checklist**

| No | Item | Guide questions/description | Section and page number in manuscript |
| --- | --- | --- | --- |
| Domain 1: Research team and reflexivity |  |  |  |
| Personal Characteristics |  |  |  |
| 1. | Interviewer/facilitator | Which author/s conducted the interview or focus group? | Methods, Setting and data collection, page 6 |
| 2. | Credentials | What were the researcher's credentials? *E.g. PhD, MD* | The primary researcher holds a master’s degree in Sociology and a master’s degree in Communication Science, additional file 1. |
| 3. | Occupation | What was their occupation at the time of the study? | The primary researcher is a PhD student, additional file 1 |
| 4. | Gender | Was the researcher male or female? | Methods, Setting and data collection, page 6 |
| 5. | Experience and training | What experience or training did the researcher have? | Methods, Setting and data collection, page 6 |
| Relationship with participants |  |  |  |
| 6. | Relationship established | Was a relationship established prior to study commencement? | No, there was no relationship established prior to the study. Participants were recruited via general practitioners, posters in a Dutch academic hospital and an item in the corporate newsletter of this hospital, Methods, Recruitment and sampling, page 5. |
| 7. | Participant knowledge of the interviewer | What did the participants know about the researcher? e*.g. personal goals, reasons for doing the research* | Participants were informed about the reasons for doing this study in the information letter. They were not informed about researchers personal goals, additional file 1. |
| 8. | Interviewer characteristics | What characteristics were reported about the interviewer/facilitator? e.g. *Bias, assumptions, reasons and interests in the research topic* | There were no characteristics of the researcher to report that might have led to bias or assumptions, additional file 1. |
| Domain 2: study design |  |  |  |
| Theoretical framework |  |  |  |
| 9. | Methodological orientation and Theory | What methodological orientation was stated to underpin the study? *e.g. grounded theory, discourse analysis, ethnography, phenomenology, content analysis* | Methods, Data analysis, page 6 |
| Participant selection |  |  |  |
| 10. | Sampling | How were participants selected? *e.g. purposive, convenience, consecutive, snowball* | Methods, Recruitment and sampling, page 5 |
| 11. | Method of approach | How were participants approached? e*.g. face-to-face, telephone, mail, email* | Methods, Recruitment and sampling, page 5 |
| 12. | Sample size | How many participants were in the study? | Methods, Setting and data collection, page 6 |
| 13. | Non-participation | How many people refused to participate or dropped out? Reasons? | Methods, Recruitment and sampling, page 5 |
| Setting |  |  |  |
| 14. | Setting of data collection | Where was the data collected? e*.g. home, clinic, workplace* | Methods, Setting and data collection, page 6 |
| 15. | Presence of non-participants | Was anyone else present besides the participants and researchers? | There were no non-participants present at the interviews, additional file 1. |
| 16. | Description of sample | What are the important characteristics of the sample? *e.g. demographic data, date* | Methods, Recruitment and sampling, page 5  Table 1, Characteristics of participants, page 22 |
| Data collection |  |  |  |
| 17. | Interview guide | Were questions, prompts, guides provided by the authors? Was it pilot tested? | Methods, Setting and data collection, page 6  Additional file 3, topic list |
| 18. | Repeat interviews | Were repeat interviews carried out? If yes, how many? | Methods, Design, page 5 |
| 19. | Audio/visual recording | Did the research use audio or visual recording to collect the data? | Methods, Setting and data collection, page 6 |
| 20. | Field notes | Were field notes made during and/or after the interview or focus group? | Methods, Setting and data collection, page 6 |
| 21. | Duration | What was the duration of the interviews or focus group? | Methods, Setting and data collection, page 6 |
| 22. | Data saturation | Was data saturation discussed? | Data were collected until no new themes emerged. Methods, Setting and data collection, page 6 |
| 23. | Transcripts returned | Were transcripts returned to participants for comment and/or correction? | The interpretations of the researcher were checked by the participant after summarizing the most important themes during and at the end of the interview. The interviews were audio-recorded and transcribed verbatim. Methods, Setting and data collection, page 6. |
| Domain 3: analysis and findings |  |  |  |
| Data analysis |  |  |  |
| 24. | Number of data coders | How many data coders coded the data? | Methods, Data analysis, page 6 |
| 25. | Description of the coding tree | Did authors provide a description of the coding tree? | The framework reflects the most important themes in the coding tree, Results, Figure 1, page 8. The codes within the themes are explained in detail throughout the results section. |
| 26. | Derivation of themes | Were themes identified in advance or derived from the data? | Methods, Data analysis, page 6 |
| 27. | Software | What software, if applicable, was used to manage the data? | Methods, Data analysis, page 6 |
| 28. | Participant checking | Did participants provide feedback on the findings? | Methods, Setting and data collection, page 6 |
| Reporting |  |  |  |
| 29. | Quotations presented | Were participant quotations presented to illustrate the themes / findings? Was each quotation identified? e*.g. participant number* | Table 3, Quotes, page 23-25 |
| 30. | Data and findings consistent | Was there consistency between the data presented and the findings? | To the authors knowledge there is consistency between the data presented and the findings, additional file 1 |
| 31. | Clarity of major themes | Were major themes clearly presented in the findings? | To the authors knowledge the major themes were clearly presented in the findings, additional file 1 |
| 32. | Clarity of minor themes | Is there a description of diverse cases or discussion of minor themes? | To the authors knowledge minor themes or diverse cases are discussed within the results section, additional file 1 |
