# Supplementary material for: Combining paid work and family care for a patient at the end of life at home: insights from a qualitative study among caregivers in the Netherlands
Source: BMC Palliat Care. 2021 Jun 24;20:93. doi: 10.1186/s12904-021-00780-9 (PMC8228921; doi:10.1186/s12904-021-00780-9)
Supplement: Supplementary file 2 — Additional file 2. Questionnaire. Questionnaire that was used for background characteristics family caregivers. [file 12904_2021_780_MOESM2_ESM.docx]

**Additional file 2 – Questionnaire**

| **Variable** | **Question** | **Response categories** | **Recodes** |
| --- | --- | --- | --- |
| Gender | What is your gender? | 1. Male 2. Female | N.a. |
| Age | What is your age? | Continuous [0-125] | N.a. |
| Education | What is the highest level of education you obtained? | 1. Basic education (including LOM, BLO, etc)  2. Pre-vocational education (LBO, LTS), VMBO  3. Mavo, VMBO-TL, ULO, MULO  4. Havo, VWO, Gymnasium, HBS, MMS  5. MBO (BOL, BBL) - level 1  6. MBO (BOL, BBL) - level 2 t/m 4  7. Higher education (HBO, WO)  8. Higher education (master or postdoctoral education)  9. None of the above | Categorized into: 1. Primary or vocational secondary education (1,2,3,5,9)  2. Secondary education (4,6)  3. Tertiary education (7,8) |
| Employment | Do you currently have paid work for at least 12 hours per week? | 1. Yes, I work as an employee 2. Yes, I am self-employed 3. No, I work less than 12 hours per week 4. No, I do not have paid work | N.a. |
| Working hours per week | How many hours do you work on average per week? | Continuous [0-168] | N.a. |
| Work sector | What is your job sector? | [open field] | 1. Business 2. Creative arts 3. Education 4. Fast-moving consumer goods 5. Healthcare and social care 6. Public services |
| Relationship to care recipient | What is your relationship to the care recipient? Care recipient is my … | 1. Partner 2. Child 3. Parent/parent-in-law 4. Brother/sister 5. Other relative 6. Neighbour 7. Friend or acquaintance 8. Other | N.a. |
| Type of illness | What type illness does the care recipient have? | 1. Chronic Obstructive Pulmonary Disease (COPD) 2. Heart failure 3. Dementia 4. Stroke (CVA) 5. Progressive neurologic disorder (e.g., ALS) 6. Incurable form of cancer, namely [open] 7. Other, namely [open] | N.a. |
| Caregiving tasks | What type of care do you provide? | 1. Emotional support 2. Help with transport 3. Assistance with doctor visits 4. Administrative support 5. Help with household chores 6. Personal care 7. Nursing care 8. Coordination and scheduling of care 9. Other | N.a. |
| Hours of care per week | How many hours of care do you provide on average per week? | Continuous [0-168] | N.a. |
| Intensity of care | What is the contact frequency with the care recipient? | 1. We live together 2. Daily contact 3. Weekly contact 4. Monthly contact | N.a. |
| Place of residence of care recipient | Where did the care recipient live in the past year? | 1. At home (same household as caregiver) 2. At home (different household to caregiver) 3. Hospital 4. Hospice 5. Residential institution 6. Nursing home 7. Other, namely [open] | N.a. |
