# Supplementary material for: Combining paid work and family care for a patient at the end of life at home: insights from a qualitative study among caregivers in the Netherlands
Source: BMC Palliat Care. 2021 Jun 24;20:93. doi: 10.1186/s12904-021-00780-9 (PMC8228921; doi:10.1186/s12904-021-00780-9)
Supplement: Supplementary file 3 — Additional file 3. Topic list for semi-structured interviews. Topic list that was used in this study. [file 12904_2021_780_MOESM3_ESM.docx]

**Additional file 3 – Topic list**

| Topic list for semi-structured interviews | |
| --- | --- |
| *Personal situation* | *(Support) needs* |
| - Work situation | - Worries |
| - Care situation | - Support from personal network |
| - Changes in work/care in past six months | - Support at work |
| - Own health | - Arrangements at work |
| *The combination of work and care* | - Support from healthcare professionals |
| - Experiences with combining work and care | - (Un)Fulfilled needs |
| - Barriers and facilitators | *Communication ^a^* |
| - Possible solutions to barriers | - Communication at work |
| - Influence of work on care | - Communication with healthcare professionals |
| - Influence of care on work | - Communication with organisations / municipalities |
| - Influence of combining work and care on own health and wellbeing | - Telephone contact with/about care recipient |
| Notes: At the end of the interview participants were asked whether all important topics were discussed and encouraged to introduce additional topics if necessary. ^a^ Communication topics were added to the topic list after analysing the first interviews as this proved to be important. | |
